# Measurement of glomerular filtration rate using endogenous d-serine clearance in living kidney transplant donors and recipients

**DOI:** 10.1016/j.eclinm.2021.101223

**Published:** 2021-12-05

**Authors:** Masataka Kawamura, Atsushi Hesaka, Ayumu Taniguchi, Shigeaki Nakazawa, Toyofumi Abe, Makoto Hirata, Ryuichi Sakate, Masaru Horio, Shiro Takahara, Norio Nonomura, Yoshitaka Isaka, Ryoichi Imamura, Tomonori Kimura

**Affiliations:** a Department of Urology, Osaka University Graduate School of Medicine, Suita, Japan; b KAGAMI Project, National Institutes of Biomedical Innovation, Health and Nutrition (NIBIOHN), Osaka, Japan; c Reverse Translational Project, Center for Rare Disease Research, National Institutes of Biomedical Innovation, Health and Nutrition (NIBIOHN), Osaka, Japan; d Department of Nephrology, Osaka University Graduate School of Medicine, Osaka, Japan; e Laboratory of Rare Disease Resource library, Center for Rare Disease Research, National Institutes of Biomedical Innovation, Health and Nutrition (NIBIOHN); f Department of Nephrology, Kansai Medical Hospital, Osaka, Japan; g Department of Renal Transplantation, Kansai Medical Hospital, Osaka, Japan

**Keywords:** d-Serine, Glomerular filtration rate, Kidney transplantation, Creatinine clearance, d-Serine clearance, GFR, glomerular filtration rate, eGFR, estimated glomerular filtration rate, mGFR, measure glomerular filtration rate, C-in, clearance of inulin, C-cre, clearance of creatinine, C-dSer, clearance of d-serine, CKD, chronic kidney disease, 2D-HPLC, two-dimensional high-performance liquid chromatography, sCre, serum creatinine, sCys, serum cystatin C, CI, confidence interaval

## Abstract

**Background:**

Endogenous molecules that provide an unbiased and a precise evaluation of kidney function are still necessary. We explored the potential of clearance of d-serine, a rare enantiomer of serine and a biomarker of kidney function, as a measure of glomerular filtration rate (GFR).

**Methods:**

This was a cross-sectional observational study of 200 living kidney transplant donors and recipients enrolled between July 2019 and December 2020 in a single Japanese center, for whom GFR was measured by clearance of inulin (C-in). Clearance of d-serine (C-dSer) was calculated based on blood and urine levels of d-serine, as measured by two-dimensional high-performance liquid chromatography. Analytical performance was assessed by calculating biases. Utilizing data from 129 participants, we developed equations for C-in based on C-dSer and C-cre using a linear regression model, and the performance was validated in 68 participants.

**Findings:**

The means of C-in and C-dSer were 66.7 and 55.7 mL/min/1.73 m^2^ of body surface area, respectively, in the entire cohort. C-dSer underestimated C-in with a proportional bias of 22.0% (95% confidence interval, 14.2–29.8%) and a constant bias of -1.24 (-5.78–3.31), whereas the proportional bias was minor to that of C-cre (34.6% [31.1–38.2%] and 2.47 (-1.18–6.13) for proportional and constant bias, respectively). Combination of C-dSer and C-cre measured C-in with an equation of 0.391 × C-dSer + 0.418 × C-cre + 3.852, which reduced the proportional bias (6.5% [-0.2–13.1%] and -4.30 [-8.87–0.28] for proportional and constant bias, respectively). In the validation dataset, this equation performed well with median absolute residual of 3.5 [2.3–4.8], and high ratio of agreement (ratios of 30% and 15% different from C-in [P_30_ and P_15_] of 98.5 [91.4–100] and 89.7 [80.0–95.2], respectively).

**Interpretation:**

The smaller proportional bias compared to that of C-cre is an advantage of C-dSer as a measure of C-in. Combinational measurement of d-serine and creatinine, two endogenous molecules, has the potential to serve as a measure of GFR with precision and minor biases and can support important clinical decisions.

**Funding:**

Japan Society for the Promotion of Science (JSPS, grant number 17H04188), Japan Agency of Medical Research and Development (AMED, JP20gm5010001), Osaka Kidney Bank (OKF19-0010), Shiseido Co., Ltd and KAGAMI Inc.

**Research in Context**

**Evidence before this study**

Current evaluation of kidney function has several limitations: a labor-intensive procedure for clearance of inulin (C-in), a major proportional bias for clearance of creatinine (C-cre), and imprecise estimation of estimated GFR (eGFR). Endogenous molecules that potentiate the precise assessment of kidney function with small biases are still necessary for important clinical decisions, including drug administration design, transplant donor selection, and chronic kidney disease (CKD) stage classification. The blood level of d-serine, a rare enantiomer of serine, reflects the prognosis of the kidney and correlates well with C-in.

**Added value of this study**

The clearance of d-serine (C-dSer) agreed well with C-in with its minor proportional bias against C-in to that with C-cre. The combination of C-dSer and C-cre measured C-in with an equation of 0.391 × C-dSer + 0.418 × C-cre + 3.852. This endogenous factor-based equation can measure the GFR with a high performance.

**Implications of all the available evidence**

The smaller proportional bias compared to that of C-cre is an advantage of C-dSer as a measure of C-in. Combinational assessment by the clearance of two endogenous molecules, d-serine and creatinine, is likely to provide precise and unbiased measures for GFR by endogenous molecules, and may support important clinical decisions, such as adjustment of drug dosages, diagnosis of CKD, and suitability as a living donor for kidney transplantation.

Alt-text: Unlabelled box

## 1 Introduction

Evaluation of kidney function is essential in daily clinical practice. The measured glomerular filtration rate (mGFR) using exogenous inulin has been considered as the gold standard of kidney function [1], though this procedure is labor intensive, time consuming, and expensive [2]. Therefore, estimation of GFR using endogenous molecules has widely been used. Clearance of creatinine (C-cre) correlates strongly with GFR, while this method has a major proportional bias and overestimates GFR due to kidney tubular secretion of creatinine into urine. The estimated GFR (eGFR), calculated using a combination of age, sex, race, and serum levels of creatinine and/or cystatin C [3,4], is convenient and useful for screen chronic kidney disease (CKD). Most equations for eGFR show relatively small bias, however, their precisions are not good enough to provide a correct estimation for the individual patient [5]. Endogenous molecules that potentiate the precise assessment of kidney function with small biases is still necessary for important clinical decisions, including drug administration design, transplant donor selection, and CKD stage classification [6,7].

d-Serine is a candidate biomarker of kidney function. l- and d-amino acids are mirror-image enantiomers of serine, and l-amino acids have been regarded as exclusively present in the human body until recently. With the development of measurement techniques and equipment [8,9], d-amino acids have been shown to exist in the body. Among d-amino acids, d-serine represented kidney function and disease activity. A higher level of d-serine in blood is associated with earlier progression to end-stage kidney diseases necessitating kidney replacement therapy [10]. Additionally, the blood level of d-serine correlates well with the measured GFR (mGFR) [11]. These studies suggest the possibility of using d-serine for the estimation of GFR.

Regarding the dynamics of d-serine in the body, d-serine is synthesized in the brain through chiral conversion from l-serine by serine racemase [12,13] or uptaken from food. d-Serine circulates the bloodstream in the body and is delivered to the kidney. d-Serine is then filtrated through the glomerulus and excreted in the urine [14]. When kidney function is impaired, the urinary excretion of d-serine decreases, and its blood level increases [11,14,15]. Since the kidney precisely regulates blood level of d-serine, we hypothesized that the kidney function could be measured by the clearance of d-serine (C-dSer).

In this cross-sectional observational study of a prospective cohort, we explored the potential agreement of C-dSer with GFR. We also developed a method to measure GFR using the combination of C-dSer and C-cre. This endogenous factor-based method can provide a more convenient method to measure GFR.

## 2 Methods

### 2.1 Study design and participants

This is a cross-sectional observational study of a prospective cohort. Participants in this prospective cohort study were adults aged 20 or older who were potential living kidney donors, post kidney donors, and kidney transplant recipients. We recruited 200 participants from three centers in Japan, and blood and urine samples were collected at Kansai Medical Hospital between July 2019 and December 2020. Analytical performance of C-dSer as a measure of GFR was assessed by calculating biases. Exclusion criteria was cases with urine output of less than 20 mL per 30 minutes. This study was conducted in compliance with the Declaration of Helsinki, the Ethical Guidelines for Medical Research Involving Human Subjects, and the Principles of the Declaration of Istanbul as outlined in the ‘Declaration of Istanbul on Organ Trafficking and Transplant Tourism’. Approval for all facilities was obtained from the Central Ethics Review Committee of Osaka University (#16330). Written informed consent for this study was obtained from all participants. This study adhered to the STROBE guidelines.

### 2.2 Clearance of inulin, creatinine and d-serine, and equations for GFR estimation

Clearance of inulin (C-in) was calculated from serum and urine inulin concentrations and urine volume using standardized methods described previously [16]. Inulin (1%; Fuji Yakuhin, Saitama, Japan) was administered intravenously using an infusion pump under fasting, medication-suspended, and hydrated conditions. The infusion rate was 300 mL/h for 0–30 min and 100 mL/h for 30–120 min. To maintain urine output during clearance measurements, participants were given 500 mL of water 30 min before the start of inulin administration and 60 mL of water at each urine collection time point. Participants urinated completely 30 minutes after the start of administration and urine was collected every 30 minutes (60, 90, and 120 minutes after the start of administration). Clearance was calculated as follows: Ux (mg/dL) × V (mL/min) / Sx (mg/dL), where x is a substrate such as inulin, creatinine, or d-serine, Ux is the urinary concentration of x, V is the flow rate, and Sx is the serum concentration of x. Each clearance was corrected for body surface area using the formula of Dubois and Dubois [17]. The mean clearance values at each time point (30–60, 60–90, 90–120) were used for analysis. Blood was collected in the middle of each urine collection time (45, 75, and 105 minutes after dosing). Serum and urine inulin concentrations were colorimetrically determined using Diacolor inulin kit (Toyobo, Osaka, Japan). The measurement is continuously calibrated using reference control material. Creatinine in serum and urine was measured enzymatically (Determiner L CRE, Hitachi Chemical, Tokyo, Japan), and serum cystatin C (sCys) was measured using an immunological turbid metric assay (Nescoat GC Cystatin C, Alfresa Pharma, Osaka, Japan). The serum creatinine and cystatin C assays were continuously calibrated using human pooled serum (L-Consera EX, Nissui Pharmaceutical, Tokyo, Japan) and cystatin C standard (Nescoat Cystatin C Standard, Alfresa Pharma), respectively. The measurements were surveyed by the Japanese Association of Medical Technologist's quality control survey project and data standardization project system (JAMTQC) as external clinical laboratory quality control. The measurement of d-serine is described below.

Estimated GFR (eGFR) was computed from equations developed by the Japanese GFR equation based on serum creatinine (sCre, eGFR_cre) [2] and sCys (eGFR_cys) [18], and CKD-EPI equation based on sCre (CKD-EPI_cre), sCys (CKD-EPI_cys), sCre-sCys (CDK-EPI_cre-cys) [4], and Caucasian and Asian pediatric and adult subjects (CAPA) equation [19]. The serum samples were taken prior to the injection of inulin in the clearance test. Detailed formulas of these equations are given in Supplementary Table 1.

### 2.3 d-Serine quantification

Blood and urine samples to measure d-serine were collected at the same time as inulin clearance measurements. The preparation of samples and quantification of serine enantiomers by a two-dimensional high-performance liquid chromatography (2D-HPLC) system were performed as previously described [8,9]. Briefly, 20-fold volumes of methanol were added to the sample and an aliquot (10 μL of the supernatant obtained from the methanol homogenate) was placed in a brown tube. After drying the solution under reduced pressure, 20 μL of 200 mM sodium borate buffer (pH 8.0) and 5 μL of fluorescence labeling reagent (40 mM 4-fluoro-7-nitro-2,1,3-benzoxadiazole (NBD-F) in anhydrous MeCN) were added, and then heated at 60°C for 2 min. An aqueous 0.1% (v/v) TFA solution (75 μL) was added, and 2 μL of the reaction mixture was subjected to the 2D-HPLC.

The enantiomers of serine were quantified using the 2D-HPLC platform. The NBD-derivatives of the amino acids were separated using a reversed-phase column (Singularity RP column, 1.0 mm i.d. × 50 mm; provided by KAGAMI Inc., Osaka, Japan) with the gradient elution using aqueous mobile phases containing MeCN and formic acid. To separately determine d- and l-serine, the fractions of serine were automatically collected using a multi-loop valve, and transferred to the enantioselective column (Singularity CSP-001S, 1.5 mm i.d. × 75 mm; KAGAMI Inc.). Then, d- and l-serine were separated in the second dimension by the enantioselective column. The mobile phases are the mixed solution of MeOH-MeCN containing formic acid, and the fluorescence detection of the NBD-amino acids was carried out at 530 nm with excitation at 470 nm using two photomultiplier tubes.

Target peaks were quantified by scaling the standard peak shape [20]. In this method, the shape of a peak was used for identification of the substrate, whereas the magnitude of the intensity was used for quantification. Prior to the quantification, peak sections of various concentrations of standard serine enantiomers (d-serine, 199-08822; l-serine, 634-25661; Fujifilm Wako Chemical Corporation, Osaka, Japan) were obtained as standard shapes for calibration. The obtained data points were multiplied by a constant so that the peak shape was the same as that of the reference peak generated based on 1 pmol injection of the serine enantiomers. Using the obtained constant, the calibration lines were made against the amounts of the injected amino acid. From the chromatogram of a sample, target shapes of serine enantiomers were identified based on the elution time and the shape of the peak. Four kinds of peak sections were assigned for each enantiomer, and the peak section, where the interference from intrinsic substances was not severe, was selected for quantification. The peak shape obtained by the standard amino acid enantiomer was superimposed to the obtained peak sections, and the magnification constant best fitted to the target peak was identified. The concentration of the target enantiomer was calculated by using the identified magnification constant and the calibration lines. The peak shape method potentiated quantification within a few seconds. The fully-automatic 2D-HPLC system required less than 10 minutes for the measurements of d-serine, including separation, identification, and quantification steps.

### 2.4 Training and validation of equations

We divided entire cohort 2:1 into training and validation dataset. In the training dataset, the least squares regression line for d-serine and creatinine clearance against inulin clearance were calculated by simple regression analysis, and the coefficient for the combined equation was calculated by multiple regression analysis. We compared the usefulness of those equations and eGFR equations in the validation dataset. The coefficients of determination (R^2^), bias, root-mean-square error (RMSE), and accuracy were calculated as a metrics for comparison. Bias of the equation was expressed as the median of the absolute value of the difference between calculated GFR and C-in. Precision was assessed as the interquartile range (IQR) for the true difference between calculated GFR and C-in. RMSE for C-in calculated using the equation was the square root of (sum of squared differences / n). Accuracy was expressed as the percentage of participants whose calculated GFR was within less than 30, 15, or 7.5% of C-in (P_30_, P_15_, and P_7_._5_). 95% confidence intervals were calculated using bootstrap resampling with a normal distribution approximation.

### 2.5 Statistical analysis

Data were expressed as mean ± standard deviation, or as count and ratio (%). The normal distribution of each variable was confirmed using qq plots, and Pearson's correlation coefficient was calculated. Comparison between methods was performed using Deming regression through the evaluation of slope and intercept of the regression line. Agreement between methods was visualized using Bland-Altman plots. Bias of each equation against C-in was compared using signed-rank test. Accuracy between equations for C-in was compared using McNemar's test. Sample size analysis was performed based on the assumption that the combination of C-dSer and C-cre agrees with GFR better than C-cre alone. Suppose that the bias of C-cre against C-in was 5, which was reduced to 80% by the combination with standard deviation for the difference was 50%, the number needed for the test based on error probability of 0.05 and power of 0.85 was 34. Since we intended to divide participants into 2:1 for training and validation set and set 20% as a margin, the total number for participants required was 163. Subgroup analyses were performed for sex, transplantation-related status, age, and GFR. Statistical significance was defined as *p* < 0.05. JMP ® pro 15.0, GraphPad Prism 5.0, and STATA 15.0 were used for statistical analyses and data visualization.

### 2.6 Role of funding source

This study was funded by Japan Society for the Promotion of Science (JSPS, grant number 17H04188), Japan Agency of Medical Research and Development (AMED, JP20gm5010001), Osaka Kidney Bank (OKF19-0010), Shiseido Co., Ltd and KAGAMI Inc. The funders had no role in study design, data collection, analysis, interpretation, or writing of the report.

## 3 Results

The background demographics of the participants are shown in Table 1 and Supplementary Table 2. The total number of eligible participants was 197, after having excluded 3 participants with low urine output. These participants consisted of 125 potential living kidney donors, 27 post-donors, and 45 transplant recipients. In the overall cohort, the mean values of serum creatinine, cystatin C, and plasma d-serine were 0.86 mg/dL, 1.1 mg/L, and 2.3 μM, respectively. The mean clearances of inulin, creatinine, and d-serine were 66.7, 98.2, and 55.7mL per minutes per 1.73m^2^ of body surface area, respectively. The levels and clearances of d-serine in plasma and serum were almost identical (Supplementary Figure S1), and we used plasma level of d-serine in the analysis.

**Table 1:** Characteristics of the participants.

	Total (*n* = 197)	Training (*n* = 129)	Validation (*n* = 68)	*P*
Participants				
Potential living donor	125 (64.5)	83 (64.3)	42 (61.8)	0.87
Post-donor	27 (13.7)	18 (14.0)	9 (13.2)	
Transplant recipient	45 (22.8)	28 (21.7)	17 (25.0)	
Age, y	60.1 ± 12.4	60.4 ± 12.7	59.5 ± 11.8	0.64
Male	84 (42.6)	56 (43.4)	22 (41.2)	0.76
Body mass index, kg/m^2^	23.2 ± 3.5	23.5 ± 3.7	22.6 ± 2.9	0.062
Body surface area, m^2^	1.63 ± 0.18	1.64 ± 0.18	1.62 ± 0.19	0.50
Diabetes	10 (5.1)	7 (5.4)	3 (4.4)	0.76
Hypertension	34 (17.3)	22 (17.1)	12 (17.7)	0.92
Hyperlipidemia	35 (17.8)	20 (15.5)	15 (22.1)	0.26
Hemoglobin, g/dL	12.7 ± 1.7	12.8 ± 1.6	12.5 ± 1.8	0.28
Serum albumin, g/dL	4.0 ± 0.3	4.0 ± 0.3	4.0 ± 0.3	0.94
Serum creatinine, mg/dL	0.86 ± 0.36	0.87 ± 0.37	0.85 ± 0.33	0.73
Serum cystatin C, mg/L	1.1 ± 0.4	1.1 ± 0.4	1.1 ± 0.4	0.56
Plasma d-serine, μM	2.3 ± 0.8	2.3 ± 0.8	2.3 ± 0.3	0.61
Inulin clearance, mL/min/1.73 m^2^	66.7 ± 20.2	64.4 ± 20.5	67.2 ± 19.9	0.80
Creatinine clearance, mL/min/1.73 m^2^	98.2 ± 30.1	98.0 ± 30.9	98.7 ± 28.7	0.87
d-Serine clearance, mL/min/1.73 m^2^	55.7 ± 16.9	55.3 ± 17.2	56.4 ± 16.4	0.66
eGFR_cre, mL/min/1.73 m^2^	67.5 ± 19.7	67.9 ± 20.8	66.8 ± 17.5	0.73
eGFR_cys, mL/min/1.73 m^2^	71.1 ± 21.6	70.1 ± 21.9	72.8 ± 21.2	0.42

Data, n (%) or mean ± SD. eGFR_cre and eGFR_cys denote creatinine- and cystatin C-based estimated glomerular filtration rates (eGFRs), respectively, based on the Japanese formula^2,18^. *P* values were given for the difference between the training and validation cohorts.

**Figure 1:**
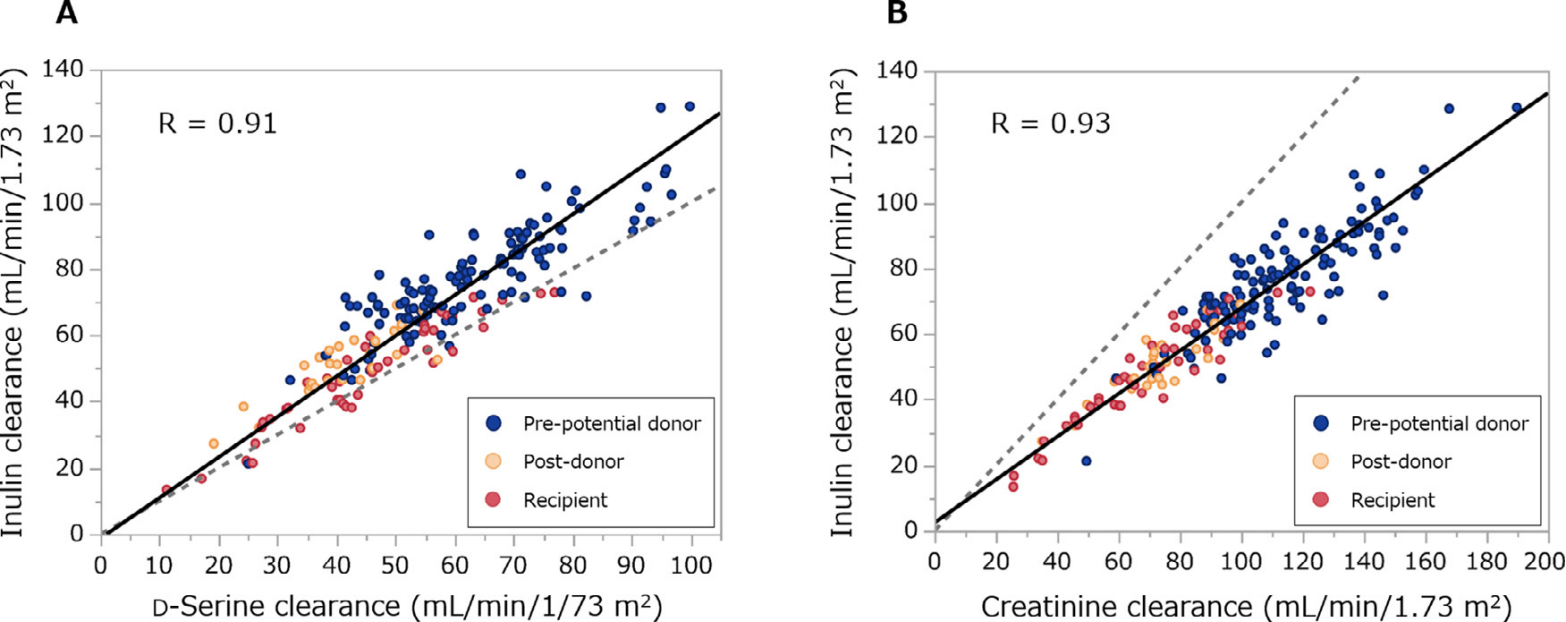
Scatter plots between clearances of inulin, d-serine and creatinine. Correlations between inulin clearance and (A) d-serine clearance and (B) creatinine clearance. Black line shows Deming regression line, and gray-dotted line shows identical line. R, Pearson's correlation coefficient.

**Figure 2:**
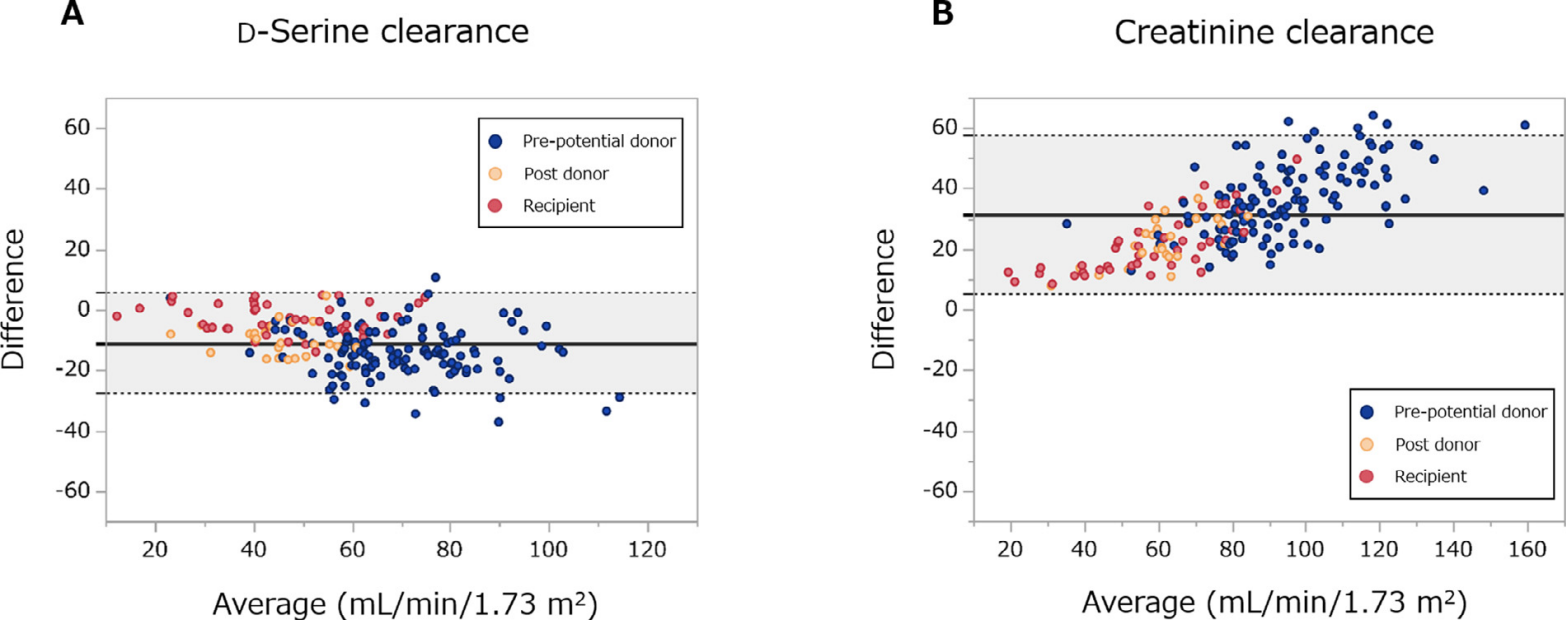
Bland-Altman plots of d-serine clearance and creatinine clearance for inulin clearance. (A) d-Serine clearance and (B) creatinine clearance. The solid black line and gray area show the mean of the difference and 95% limits of agreement.

In order to examine whether C-dSer is reliable as a measure of C-in, we first analyzed the analytical performance (Figure 1). In the total cohort population, C-dSer was significantly and strongly correlated with C-in (R = 0.91, *P* < 0.0001) like C-cre (R = 0.93). C-dSer underestimated C-in with a proportional bias of 22.0% (95% Confidence interval, 14.2–29.8%) and a constant bias of -1.24 (-5.78–3.31), whereas this proportional bias was minor to that of C-cre (34.6% [31.1–38.2%] and 2.47 (-1.18–6.13) for a proportional and a constant bias, respectively) Table 2. The Brand-Altman plot confirmed the smaller bias of C-dSer in the measurement of C-in than that of C-cre (Figure 2).

**Table 2:** Comparison between clearances of d-serine and creatinine as a surrogate for inulin clearance.

	Slope (95% CI)	Intercept (95% CI)
d-Serine clearance	1.220 (1.142–1.298)	-1.24 (-5.78–3.31)
Creatinine clearance	0.654 (0.618–0.689)	2.47 (-1.18–6.13)

Slopes and intercepts of Deming regression lines are shown. CI, confidence interval.

Since the small proportional bias in the measurement of GFR is the advantage of C-dSer, we developed C-dSer-based methods to measure GFR. For this purpose, we divided entire cohort into 129 participants for training cohort and 68 for validation (2:1) randomly (Table 1). The training and validation dataset were indifferent in the distributions and proportions of all variables. Table 3 shows the coefficients of the equation calculated based on training dataset by regression analysis for C-dSer, C-cre, and their combination. The values and the regression lines for each equation are shown in the Supplementary Figure S2. The equation for C-in based on the combination of C-dSer and C-cre was expressed as: 0.391 × C-dSer + 0.418 × C-cre + 3.852. This equation greatly reduced the proportional bias (6.5% [-0.2–13.1%] and -4.30 [-8.87–0.28] for proportional and constant bias, respectively; Supplementary Table 3).

**Table 3:** Coefficients of each equation based on clearances of d-serine and creatinine determined in the training dataset.

Parameter for equation	Equation for estimating GFR	95% CI of coefficient			
		d-Serine clearance	Creatinine clearance		Intercept
d-Serine clearance	1.085 × C-dSer + 6.434	0.998–1.172	−		1.419–11.449
Creatinine clearance	0.618 × C-cre + 5.869	−	0.577–0.660		1.606–10.133
Combination of d-serine and creatinine clearances	0.391 × C-dSer + 0.418 × C-cre + 3.852	0.210–0.572	0.318–0.518		-0.255–7.959

C-dSer and C-cre, clearances of d-serine and creatinine, respectively. CI, confidence interval

**Figure 3:**
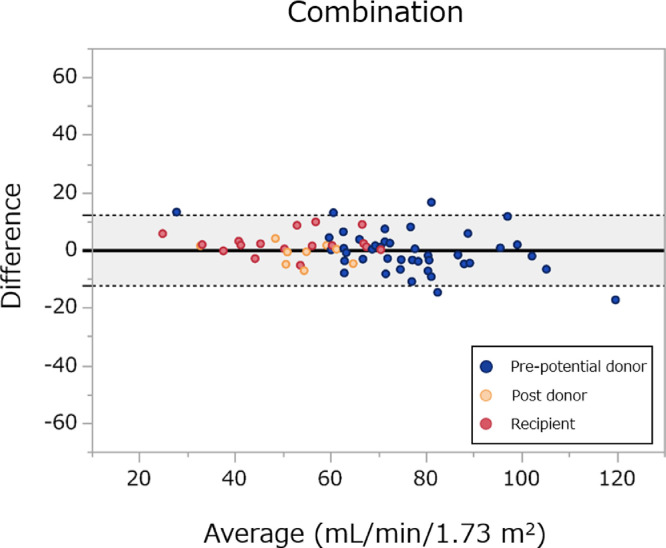
Bland-Altman plot of equation for inulin clearance in the validation cohort. Equation is based on the combination of d-serine clearance and creatinine clearance. The solid black line and gray area show the mean of the difference and 95% limits of agreement.

We then examined the performance of the established equation in validation dataset (Table 4). C-dSer performed very well, which became further better when C-dSer was used in combination with C-cre. R^2^ for combination of C-dSer and C-cre, C-dSer alone, C-cre alone were 0.90, 0.83, and 0.87, respectively. The combined equation of C- dSer and C-cre was the least biased with median absolute residual for C-in of 3.5 [95% confidence interval, 2.3–4.8] mL/min/ 1.73 m^2^. This bias was significantly less than any other equations, including C-cre (4.5 [3.2–5.7]; *P* = 0.002). Regarding precision and RMSE, combined equation was the best; it had the lowest IQR, 6.5 [4.8–9.6] and RMSE, 6.2 [5.1–7.5]. In terms of accuracy, the combined equation agreed well with C-in accurately (P_15_, 89.7 [80.0–95.2]). The combined equation provided the better accuracy of prediction within 30, 15, and 7.5% of C-in than other equations. For P_15_ and P_7·5_, the accuracy of combined equation was superior to that of C-cre (*P* = 0.04 and 0.02, respectively). The Brand-Altman plot showed the best agreement between the combination equation and C-in, which was consistent across GFR values and least biased (Figure 3, Supplementary Figure S3). Performance of the equations were consistent across the subgroups defined by sex, transplantation-related status, age, and GFR (Supplementary Figure S4). Similar performance was observed for the equation based on combination of clearances of serum d-serine and creatinine (Supplementary Figure S5, Supplementary Table 4–6). Overall, the equations based on combination of clearances of intrinsic factors, d-serine and creatinine, enabled the measurement of GFR with lower bias and higher precision.

**Table 4:** Performance of equations in the validation dataset

Equations	R^2^	Bias (95% CI)	IQR (95% CI)	RMSE (95% CI)	P30% (95% CI)	P15% (95% CI)	P7.5% (95% CI)
Combination	0.90	3.5 (2.3–4.8)	6.5 (4.8–9.6)	6.2 (5.1–7.5)	98.5 (91.4–100.0)	89.7 (80.0–95.2)	64.7 (52.8–75.0)
d-Serine clearance	0.83	5.6 (3.5–7.2)*	10.7 (7.1–14.8)	8.2 (6.9–9.6)	97.1 (89.3–99.8)	79.4 (68.2–87.4)*	45.6 (34.3–57.4)*
Creatinine clearance	0.87	4.5 (3.2–5.7)*	8.9 (6.5–12.8)	7.3 (6.0–8.7)	97.1 (89.3–99.8)	83.8 (73.1–90.9)*	51.5 (39.8–63.0)*
eGFR_cys	0.65	7.2 (5.6–11.9)*	16.6 (11.4–19.9)	12.8 (10.6–15.4)	88.2 (78.2–94.2)*	58.8 (47.0–69.8)*	36.8 (26.3–48.7)*
eGFR_cre	0.55	6.9 (5.3–9.4)*	14.1 (10.2–18.8)	13.7 (10.4–17.3)	85.3 (74.8–92.0)*	64.7 (52.8–75.0)*	33.8 (23.7–45.7)*

The combination denotes an equation based on a combination of clearances of d-serine and creatinine. eGFR_cre and eGFR_cys denote creatinine- and cystatin C-based estimated glomerular filtration rates (eGFRs), respectively, based on the Japanese formula.^2,18^ Performances of conventionally-used eGFRs were shown as references. Bias, absolute value of residual. IQR, interquartile range of difference; RMSE, root mean square error. Accuracy was calculated as the ratios that differed from inulin clearance by less than 30%, 15%, and 7.5% (P_30_, P_15_, and P_7.5_). CI, confidence interval. *Statistically significant versus combination equation.

## 4 Discussion

In the present study, we demonstrated that C-dSer is a reliable endogenous measure of GFR. C-dSer correlates well with GFR and has the advantage of a small proportional bias for the measurement of C-in over C-cre. An equation based on C-dSer enables the precise measurement of the GFR, and the equation based on the combination of C-cre and C-dSer achieved precise GFR measurement with minor biases. Measurement of endogenous d-serine will provide key information for precision medicine.

Measurement of kidney function is essential in daily clinical practice. Precise and unbiased measurement of kidney function is particularly crucial in the decision of the following situations: adjustment of drug dosages [21], diagnosis of CKD [22], and suitability as a living donor for kidney transplantation [23]. C-in is the gold standard measurement of GFR [1], and additional methods using a variety of radioactive (^99^mTc-DTPA, ^125^I-iothalamate, and ^51^Cr-EDTA) and nonradioactive (iohexol and iothalamate) tracers have been developed thus far [24]. These methods require the administration of exogenous substances, which imposes a considerable burden on the patient and labor on the examiner. Therefore, precise and unbiased estimation of GFR based on endogenous markers have been awaited. On the other hand, eGFR equations have been developed using endogenous markers such as creatinine and cystatin C, which have been shown to correlate well with GFR [3]. This approach has made it possible to estimate renal function based on a single blood test result, age, sex, and race [25]. While this method is widely used for screening of renal function because of its simplicity, it has the limitations in terms of precision [26]. In particular, it is known that there is a relatively large difference from GFR in populations with good renal function [7], and the use of eGFR alone is not recommended in key clinical situations such as to determine the suitability of living donors for kidney transplantation [27]. C-dSer may solve this clinical problem. In the measurement of C-dSer, urine samples can be collected flexibly, i.e., 30 min or 24 h, which is another advantage of measuring clearances of endogenous molecules.

d-Serine may provide a key information for GFR. One of the major determinants of d-serine dynamics is the blood flow to the kidney. Once GFR decreases, the kidneys are unable to filter d-serine at glomeruli and therefore it is not excreted through the urine [11]. As a result, we assume that clearance of d-serine was highly correlated with GFR, which mainly reflects blood flow to the kidney. Another determinant of d-serine dynamics is tubular reabsorption. About 40 % of d-serine filtered at glomerular is reabsorbed at the proximal part of tubules [15]. This is in contrast to creatinine, which has a small amount of tubular secretion. These phenomena were reflected in higher level of C-cre, and lower level of C-dSer, than that of GFR. Since d-serine and creatinine are oppositely handled at tubules, their combination may have provided additional information that lead to the determination of GFR. The dynamics of d-serine are affected neither by circadian rhythm [28] nor food restriction, the latter of which may be due to the synthesis [12,13]. These features of d-serine are favorable as a biomarker for the kidney function. Additionally, measuring clearance can potentially offset the unknown factors that influence the levels of molecules in blood and urine. Thus, C-dSer may relatively be unbiased as a measure of C-in.

The combined equation provided precise measurement with minor biases of GFR. Bias for C-in was 3.5 mL/min/1.73 m^2^ in the validation dataset, which was lowest among those of equations based on endogenous molecules. Accuracy of the combination equation was 98.5% for P_30_ and 89.7% for P_15_. These results were comparable to those reported in previous studies using iohexol to calculate mGFR (68–100% for P_30_ and 32–72% for P_10_) [29].

There are limitations to this study. First, the cohort was small and the collection of blood and urine was done at a single institution. Further validation is needed by expanding the cohort through multicenter studies. Second, our study cohort consisted of only potential living kidney donors, post donors, and transplant recipients, and did not consist of patients with chronic kidney disease, including glomerulonephritis and vasculitis. Additionally, the dominancy of potential living kidney donors with relatively favorable kidney function in this cohort may limit the evaluation in the rest of the cohort. Third, it is possible that our findings are database-specific, as the cohort consisted only of Japanese. To verify the result, it will be necessary to measure d-serine in multiple racial groups.

In conclusion, the combination of C-dSer and C-cre can measure the GFR with precision and minor biases. The current method using endogenous molecules may reduce clinical burdens for measuring GFR. The new method is precise across the tested range of GFR and has the potential to facilitate key clinical decisions in various clinical situations.

## 5 Contributors

Conceptualization, TK; data curation & formal analysis, MK, TK; funding acquisition; YI, TK; investigation, MK, AH, AT, SN, TA, MHi, RS, MHo, ST, RI and TK; project administration, NN, YI, RI, TK; validation, MK, AH, AT, SN, TA, MHi, RS, MHo, ST, NN, YI, RI and TK; visualization & writing – original draft, MK, TK; writing – review & editing, MK, AH, TK. All authors had full access to all the data, approved the manuscript, and are responsible for the decision to submit for publication.

## Declaration of Competing Interest

A part of this study was funded by Shiseido Co., Ltd and KAGAMI Inc. TK has an equity in KAGAMI Inc. TK is an inventor on issued and applied patents (WO2020080484A1, PCT/JP2020/048977), which are related with this work. All other authors declare no competing interests.

## Appendix Supplementary materials

Image, application 1

Image, application 2

## References

[1] Smith HW.*The kidney: structure and function in health and disease*: Oxford University Press, USA; 1951.

[2] Matsuo S, Imai E, Horio M (2009). Revised equations for estimated GFR from serum creatinine in Japan. Am J Kidney Dis.

[3] Levey AS, Stevens LA, Schmid CH (2009). A new equation to estimate glomerular filtration rate. Ann Intern Med.

[4] Inker LA, Schmid CH, Tighiouart H (2012). Estimating glomerular filtration rate from serum creatinine and cystatin C. N Engl J Med.

[5] Porrini E, Ruggenenti P, Luis-Lima S (2019). Estimated GFR: time for a critical appraisal. Nat Rev Nephrol.

[6] Delanaye P, Schaeffner E, Ebert N (2012). Normal reference values for glomerular filtration rate: what do we really know?. Nephrol Dial Transplant.

[7] González-Rinne A, Luis-Lima S, Escamilla B (2019). Impact of errors of creatinine and cystatin C equations in the selection of living kidney donors. Clin Kidney J.

[8] Miyoshi Y, Hamase K, Tojo Y, Mita M, Konno R, Zaitsu K. (2009). Determination of D-serine and D-alanine in the tissues and physiological fluids of mice with various D-amino-acid oxidase activities using two-dimensional high-performance liquid chromatography with fluorescence detection. J Chromatogr B Analyt Technol Biomed Life Sci.

[9] Hamase K, Miyoshi Y, Ueno K (2010). Simultaneous determination of hydrophilic amino acid enantiomers in mammalian tissues and physiological fluids applying a fully automated micro-two-dimensional high-performance liquid chromatographic concept. J Chromatogr A.

[10] Kimura T, Hamase K, Miyoshi Y (2016). Chiral amino acid metabolomics for novel biomarker screening in the prognosis of chronic kidney disease. Sci Rep.

[11] Hesaka A, Sakai S, Hamase K (2019). D-Serine reflects kidney function and diseases. Sci Rep.

[12] Miya K, Inoue R, Takata Y (2008). Serine racemase is predominantly localized in neurons in mouse brain. J Comp Neurol.

[13] Wolosker H, Mori H. (2012). Serine racemase: an unconventional enzyme for an unconventional transmitter. Amino Acids.

[14] Kimura T, Hesaka A, Isaka Y. (2020). D-Amino acids and kidney diseases. Clin Exp Nephrol.

[15] Sasabe J, Suzuki M, Miyoshi Y (2014). Ischemic acute kidney injury perturbs homeostasis of serine enantiomers in the body fluid in mice: early detection of renal dysfunction using the ratio of serine enantiomers. PloS one.

[16] Wesson LG. (1969).

[17] Du Bois D, Du Bois EF (1916). A formula to estimate the approximate surface area if height and weight be known. Nutrition (Burbank, Los Angeles County, Calif).

[18] Horio M, Imai E, Yasuda Y, Watanabe T, Matsuo S. (2013). GFR estimation using standardized serum cystatin C in Japan. Am J Kidney Dis.

[19] Grubb A, Horio M, Hansson LO (2014). Generation of a new cystatin C-based estimating equation for glomerular filtration rate by use of 7 assays standardized to the international calibrator. Clin Chem.

[20] Hamase K, Ikeda T, Ishii C (2018). Determination of Trace Amounts of Chiral Amino Acids in Complicated Biological Samples Using Two-Dimensional High-Performance Liquid Chromatography with an Innovative “Shape-Fitting” Peak Identification/Quantification Method. CHROMATOGRAPHY.

[21] Drugs Vincent J., Kidneys the (2017). Clinical Pharmacology Perspectives. Clin Pharmacol Ther.

[22] Webster AC, Nagler EV, Morton RL, Masson P. (2017). Chronic Kidney Disease. Lancet.

[23] MacPhee IAM. (2018). Measured Versus Estimated Glomerular Filtration Rate in the Assessment of Living Kidney Donors: eGFR Has Its Limitations. Transplantation.

[24] White CA, Akbari A, Allen C (2021). Simultaneous glomerular filtration rate determination using inulin, iohexol, and (99m)Tc-DTPA demonstrates the need for customized measurement protocols. Kidney international.

[25] Schaeffner ES, Ebert N, Delanaye P (2012). Two novel equations to estimate kidney function in persons aged 70 years or older. Ann Intern Med.

[26] da Silva Selistre L, Rech DL, de Souza V, Iwaz J, Lemoine S, Dubourg L. (2019). Diagnostic Performance of Creatinine-Based Equations for Estimating Glomerular Filtration Rate in Adults 65 Years and Older. JAMA Intern Med.

[27] Delmonico F. (2005). A Report of the Amsterdam Forum On the Care of the Live Kidney Donor: Data and Medical Guidelines. Transplantation.

[28] Morikawa A, Hamase K, Miyoshi Y, Koyanagi S, Ohdo S, Zaitsu K. (2008). Circadian changes of D-alanine and related compounds in rats and the effect of restricted feeding on their amounts. J Chromatogr B Analyt Technol Biomed Life Sci.

[29] Soveri I, Berg UB, Björk J (2014). Measuring GFR: a systematic review. Am J Kidney Dis.

