# Supplementary material for: Measurement of glomerular filtration rate using endogenous d-serine clearance in living kidney transplant donors and recipients
Source: eClinicalMedicine. 2021 Dec 5;43:101223. doi: 10.1016/j.eclinm.2021.101223 (PMC8654629; doi:10.1016/j.eclinm.2021.101223)
Supplement: Supplementary file 2 [file mmc2.pdf]

Supplementary information

Determination of glomerular filtration rate using endogenous D-serine clearance in living kidney transplant donors and recipients

Masataka Kawamura, Atsushi Hesaka, Ayumu Taniguchi, Shigeaki Nakazawa, Toyofumi Abe, Maiko Nakane, Masashi Mita, Masaru Horio, Shiro Takahara, Norio Nonomura, Yoshitaka Isaka, Ryoichi Imamura, Tomonori Kimura

6 Supplementary tables
5 Supplementary figures

Supplementary Table 1: **List of equations for estimated glomerular filtration rate (eGFR) used in this study.**

Equations	Serum creatinine	Serum cystatin C	Equation for estimating GFR
Japanese eGFR_cre			
Female of male			$194 \times SCr^{-1.094} \times age^{-0.287}$ [×0.739 if female]
Japanese eGFR_cys			
Female			$(104 \times SCys^{-1.019} \times 0.996^{Age} \times 0.929) - 8$
Male			$(104 \times SCys^{-1.019} \times 0.996^{Age}) - 8$
CKD-EPI_cre			
Female	≤0.7		$144 \times (SCr / 0.7)^{-0.329} \times 0.993^{Age}$
Female	>0.7		$144 \times (SCr / 0.7)^{-1.209} \times 0.993^{Age}$
Male	≤0.9		$141 \times (SCr / 0.9)^{-0.411} \times 0.993^{Age}$
Male	>0.9		$141 \times (SCr / 0.9)^{-1.209} \times 0.993^{Age}$
CKD-EPI_cys			
Female of male		≤0.8	$133 \times (SCys / 0.8)^{-0.499} \times 0.996^{Age}$ [×0.932 if female]
Female of male		>0.8	$133 \times (SCys / 0.8)^{-1.328} \times 0.996^{Age}$ [×0.932 if female]
CKD-EPI_cre-cys			
Female	≤0.7	≤0.8	$130 \times (SCr / 0.7)^{-0.248} \times (SCys / 0.8)^{-0.375} \times 0.995^{Age}$
		>0.8	$130 \times (SCr / 0.7)^{-0.248} \times (SCys / 0.8)^{-0.711} \times 0.995^{Age}$
Female	>0.7	≤0.8	$130 \times (SCr / 0.7)^{-0.601} \times (SCys / 0.8)^{-0.375} \times 0.995^{Age}$
		>0.8	$130 \times (SCr / 0.7)^{-0.601} \times (SCys / 0.8)^{-0.711} \times 0.995^{Age}$
Male	≤0.9	≤0.8	$135 \times (SCr / 0.9)^{-0.207} \times (SCys / 0.8)^{-0.375} \times 0.995^{Age}$
		>0.8	$135 \times (SCr / 0.9)^{-0.207} \times (SCys / 0.8)^{-0.711} \times 0.995^{Age}$
Male	>0.9	≤0.8	$135 \times (SCr / 0.9)^{-0.601} \times (SCys / 0.8)^{-0.375} \times 0.995^{Age}$
		>0.8	$135 \times (SCr / 0.9)^{-0.601} \times (SCys / 0.8)^{-0.711} \times 0.995^{Age}$
CAPA			$130 \times SCys^{-1.069} \times age^{-0.117} - 7$

SCre, serum creatinine; SCys, serum cystatin C.

Supplementary Table 2: **Additional characteristics of the participants.**

	**Total (*n* = 197)**	**Training (*n* = 129)**	**Validation (*n* = 68)**	***P***
Systolic blood pressure, mmHg	129.0 ± 17.4	128.8 ± 17.2	129.3 ± 17.7	0.86
Diastolic blood pressure, mmHg	77.4 ± 11.4	77.5 ± 11.3	77.2 ± 11.8	0.87
Serum creatinine, mg/dL				
Female	0.71 ± 0.21	0.70 ± 0.22	0.72 ± 0.20	0.64
Male	1.08 ± 0.40	1.09 ± 0.41	1.04 ± 0.38	0.58
Serum D-serine, μM	2.4 ± 0.9	2.5 ± 0.9	2.4 ± 0.8	0.54
Serum protein, g/dL	6.7 ± 0.5	6.7 ± 0.5	6.6 ± 0.5	0.27
Serum UN, mg/dL	15.2 ± 4.7	15.1 ± 4.9	15.4 ± 4.2	0.59
Serum Na, mEq/L	138.6 ± 2.2	138.6 ± 2.3	138.7 ± 2.0	0.71
Serum K, mEq/L	4.0 ± 0.3	4.1 ± 0.3	4.0 ± 0.4	0.52
Serum Cl, mEq/L	103.8 ± 2.7	103.7 ± 2.7	104.0 ± 2.6	0.43
Serum adjusted Ca, mg/dL	9.3 ± 0.5	9.3 ± 0.5	9.4 ± 0.5	0.23
Serum iP, mg/dL	3.1 ± 0.6	3.1 ± 0.6	3.2 ± 0.6	0.21
Serum AST, U/L	21.5 ± 12.7	21.7 ± 15.0	21.2 ± 6.7	0.80
Serum ALT, U/L	18.0 ± 12.0	17.5 ± 11.4	19.2 ± 13.1	0.33
Serum T-Cho, mg/dL	196.4 ± 38.9	193.9 ± 40.8	201.2 ± 35.0	0.21
Serum TG, mg/dL	125.9 ± 98.4	119.4 ± 94.2	138.3 ± 105.4	0.20
Serum β 2-microglobulin, μg/L	2.1 ± 1.2	2.2 ± 1.2	2.1 ± 1.2	0.65
Blood WBC, ×103 /μL	5.5 ± 1.6	5.6 ± 1.5	5.2 ± 1.6	0.15
Blood PLT, ×103 /μL	228.0 ± 60.1	230.4 ± 64.8	223.6 ± 52.6	0.46
CKD-EPI_cre, mL/min/1.73 m^2	85.3 ± 20.4	84.9 ± 21.7	86.1 ± 18.0	0.69
CKD-EPI_cys, mL/min/1.73 m^2	78.1 ± 25.0	77.0 ± 25.4	80.2 ± 24.3	0.40
CKD-EPI_cre-cys, mL/min/1.73 m^2	80.4 ± 22.7	79.7 ± 23.4	81.7 ± 21.4	0.55
CAPA, mL/min/1.73 m^2	74.8 ± 23.4	73.8 ± 23.7	76.6 ± 22.9	0.43

Data are mean ± SD. *P* values were given for the difference between the training and validation cohorts. Estimated glomerular filtration rates (eGFRs) were calculated as reported[4,19].

Supplementary Table 3: **Comparison of equations based on clearances of D-serine and creatinine for inulin clearance in the training dataset.**

	**Slope (95%CI)**	**Intercept (95%CI)**
Combination of D-serine and creatinine clearances	1.065 (0.998–1.131)	-4.30 (-8.87–0.28)
D-Serine clearance	1.109 (1.020–1.197)	-7.22 (-13.33–-1.12)
Creatinine clearance	1.076 (1.004–1.118)	-5.04 (-10.03–-0.04)

Slopes and intercepts of Deming regression lines are shown. CI, confidence interval.

Supplementary Table 4: **Coefficients of equation for inulin clearance using clearances of serum D-serine and creatinine in the training dataset.**

Parameter for equation	Equation for GFR	95% CI of coefficient		
		D-Serine clearance	Creatinine clearance	Intercept
Combination of serum D-serine and creatinine clearances	0.347 × C-Dser + 0.447 × C-cre + 4.530	0.150–0.543	0.341–0.552	0.372–8.688

C-DSer and C-cre, clearances of D-serine and creatinine, respectively. GFR, glomerular filtration rate; CI, confidence interval.

Supplementary Table 5: **Evaluation of equation based on clearances of serum D-serine and creatinine for inulin clearance in the training dataset.**

	**Slope (95% CI)**	**Intercept (95% CI)**
Combination of serum D-serine and creatinine clearances	1.068 (1.000–1.136)	-4.52 (-9.22–0.19)

Slopes and intercepts of Deming regression lines are shown. CI, confidence interval.

Supplementary Table 6: **Performance of equations in the validation dataset using clearance of serum D-serine and other formulas for estimated glomerular filtration rate (eGFR).**

Equations	R^2	Bias (95% CI)	IQR (95% CI)	RMSE (95% CI)	P30% (95% CI)	P15% (95% CI)	P7.5% (95% CI)
Serum combination	0.90	3.0 (2.5–5.5)	6.2 (4.2–9.0)	6.4 (5.3–7.7)	98.5 (91.4–100.0)	88.2 (78.2–94.2)	61.8 (49.9–72.4)
CKD-EPI_cre	0.55	18.0 (14.7–20.5)	16.3 (11.7–23.2)	13.6 (11.0–17.2)	54.4 (42.7–65.7)	26.5 (17.4–38.1)	11.8 (5.8–21.8)
CKD-EPI_cys	0.61	11.4 (6.6–16.2)	20.5 (15.0–27.9)	15.2 (12.9–17.7)	69.1 (57.3–78.9)	47.1 (35.7–58.8)	23.5 (14.9–35.0)
CKD-EPI_cre-cys	0.67	13.1 (10.0–15.7)	15.0 (10.2–22.3)	12.5 (10.1–15.7)	61.8 (49.9–72.4)	35.3 (25.0–47.2)	22.1 (13.7–33.4)
CAPA	0.60	12.1 (10.7–13.6)	17.0 (14.1–20.9)	12.6 (10.1–15.9)	76.5 (64.6–85.9)	54.4 (41.9–66.6)	38.2 (26.7–50.8)

Serum combination, equation based on a combination of clearances of serum D-serine and creatinine. Estimated glomerular filtration rates (eGFRs) were calculated as reported.[4,19] Bias, absolute value of residual. IQR, interquartile range of difference; RMSE, root mean square error. Accuracy was calculated as the percentage of estimates that differed from inulin clearance by less than 30%, 15%, and 7.5% (P_{30}, P_{15}, and $P_{7.5}$). CI, confidence interval.

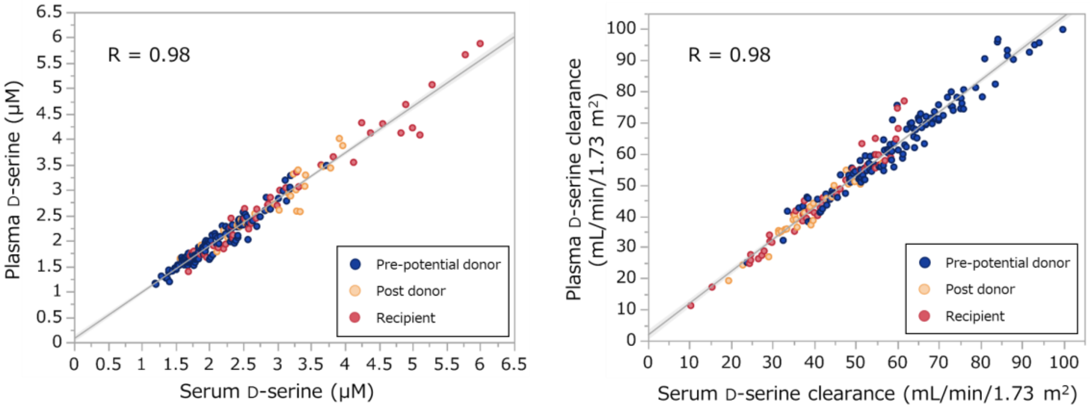

Supplementary Figure S1: **Blood levels and clearances of D-serine in plasma and serum.**

Gray line shows the linear regression and gray area represents a 95% confidence interval. R, Pearson's correlation coefficient.

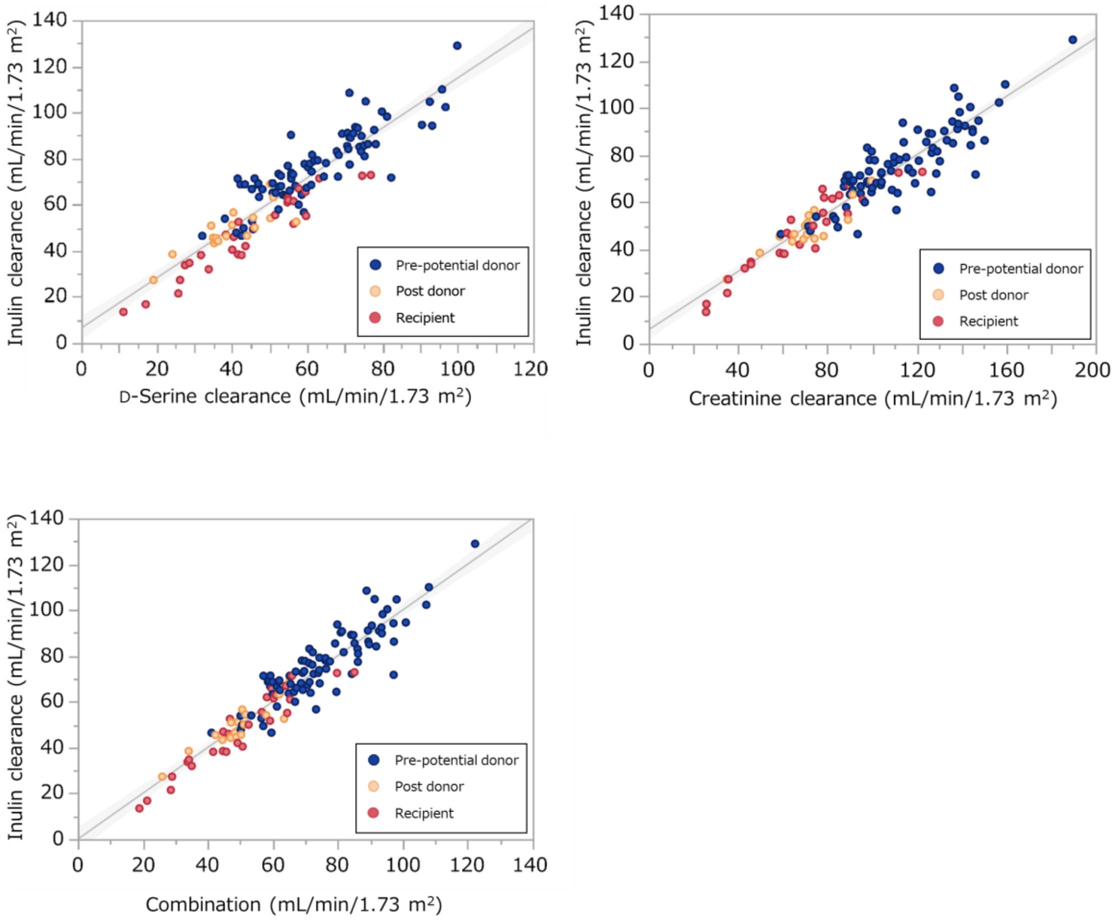

Supplementary Figure S2: **Regression line for calculating the prediction equations based on clearances of plasm D-serine and creatinine in the training dataset.**
Grey area represents a 95% confidence interval.

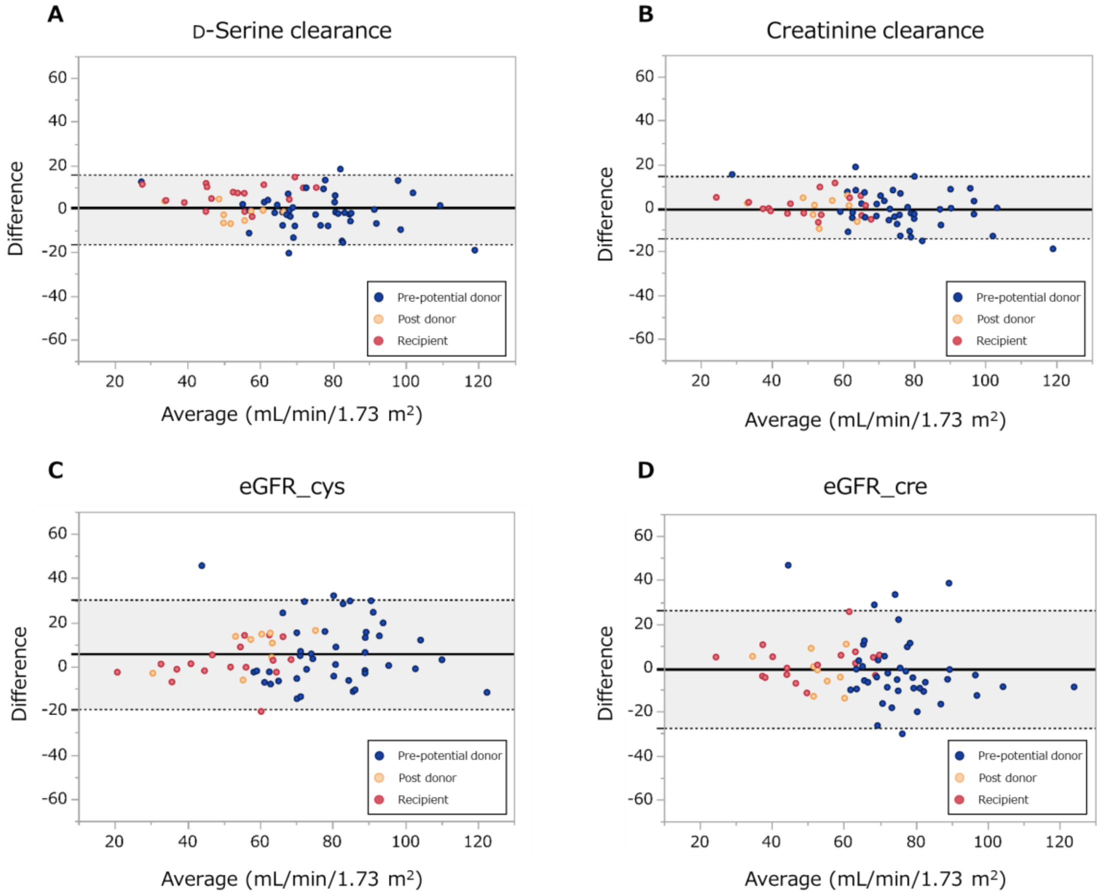

Supplementary Figure S3: **Bland-Altman plots of equations for inulin clearance in the validation dataset.**

(A-B) Equations based on (A) the D-serine clearance, (B) creatinine clearance, and (C-D) estimated glomerular filtration rate (eGFR) based on (C) creatinine (eGFR_cre), and (D) cystatin C (eGFR_cys) by Japanese formura.[2,18] The solid black line and gray area show the mean of the difference and 95% limits of agreement.

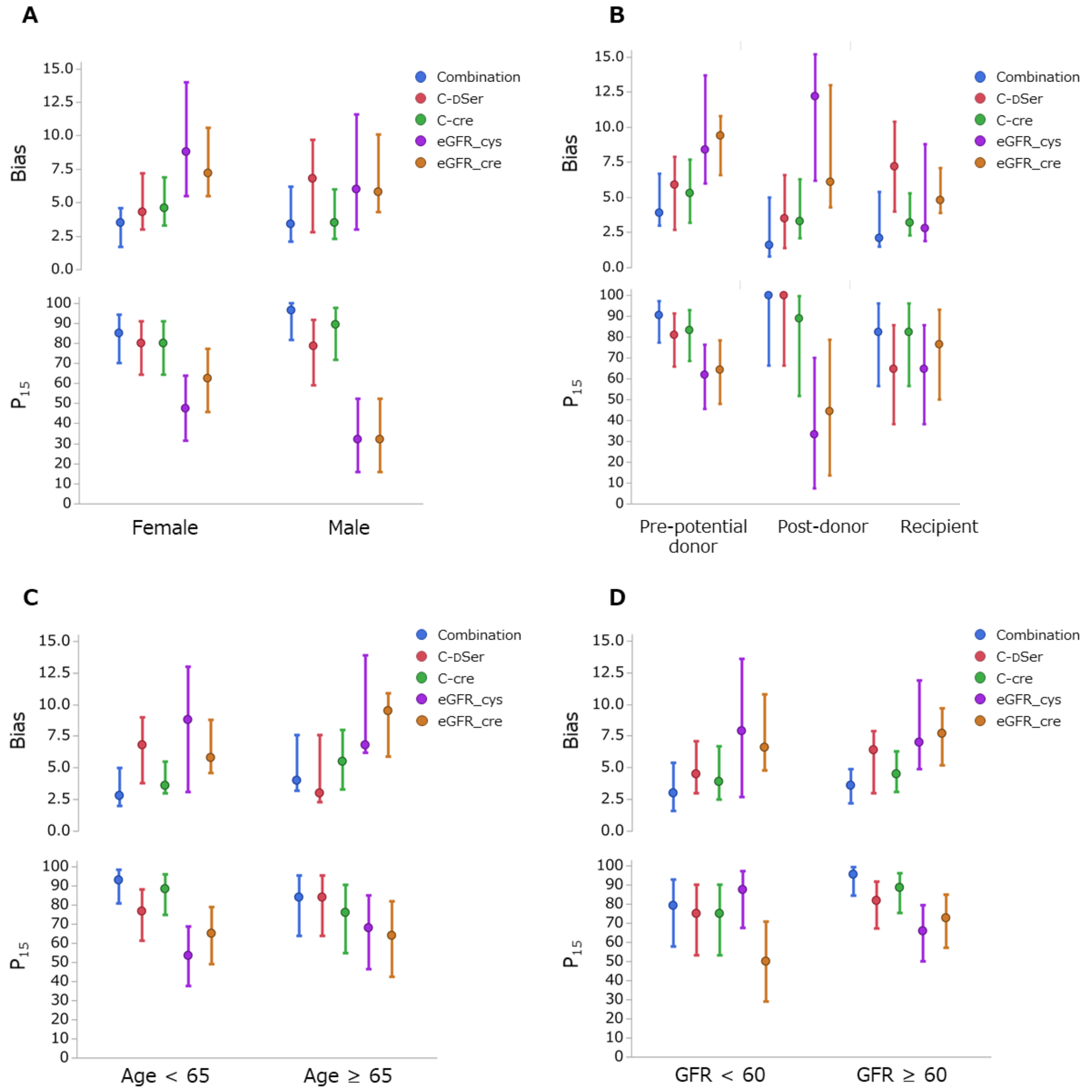

Supplementary Figure S4: **Subgroup analysis of bias and P_{15}.**

(A) Sex, (B) transplantation-related status, (C) age, and (D) glomerular filtration rate (GFR). Bars indicate 95% confidence intervals. C-DSer and C-cre, clearances of D-serine and creatinine, respectively; eGFR_cre and eGFR_cys, creatinine- and cystatin C-based estimated GFRs (eGFRs), respectively, based on the Japanese formula.[2,18]

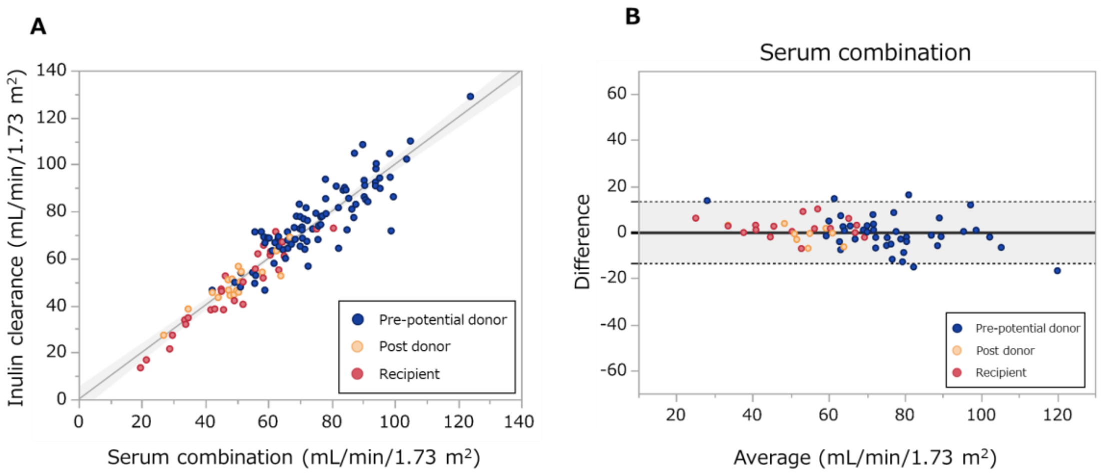

Supplementary Figure S5: **Equation using combination of clearances of serum D-serine and creatinine and its agreement.**

(A) Regression line for calculating the prediction equation based on a combination of clearances of serum D-serine and creatinine in the training dataset. Grey area represents a 95% confidence interval. (B) Bland-Altman plot of equation for inulin clearance in the validation cohort. Equation is based on the combination of serum D-serine clearance and creatinine clearance. The solid black line and gray area show the mean of the difference and 95% limits of agreement.
